# Diurnal variation of the distance between cranium and the third lumbar vertebra and its implications for craniospinal irradiation

**DOI:** 10.1016/j.phro.2025.100760

**Published:** 2025-04-08

**Authors:** Annele Heikkilä, Maija Rossi, Antti Vanhanen, Tuomas Koivumäki, Michiel Postema, Eeva Boman

**Affiliations:** a Department of Biomedical Technology, Faculty of Medicine and Health Technology, Tampere University, Korkeakoulunkatu 8, 33720 Tampere, Finland; b Department of Clinical Physiology, Nuclear Medicine and Medical Physics, Tampere University Hospital, Wellbeing Services County of Pirkanmaa, P.O. Box 2000, 33521, Tampere, Finland; c Department of Oncology, Tampere University Hospital, Wellbeing Services County of Pirkanmaa, P.O. Box 2000, 33521, Tampere, Finland; d Department of Medical Physics, Hospital Nova of Central Finland, Wellbeing Services County of Central Finland, Hoitajantie 3, 40620 Jyväskylä, Finland; e School of Electrical and Information Engineering, University of the Witwatersrand, Johannesburg, Jan Smutslaan 1, 2050 Braamfontein, South Africa

**Keywords:** Craniospinal radiotherapy, Circadian height variation, Diurnal changes in spinal length, Setup errors in CSI

## Abstract

The spine shortens during the day because of gravity. This study quantified the effect of treatment fraction timing on spinal length in 13 craniospinal irradiation patients. The distance deviation from the base of skull to the third lumbar vertebra in daily planar kilovoltage setup images compared to the treatment planning computed tomography image was determined. The time deviation between the treatment fraction and planning computed tomography image was registered. A distance decrease of 0.8±0.2 mm/hour was observed. Timing the treatment fractions within two hours of the planning imaging session is advisable to minimise the potential dosimetric impact of diurnal variations.

## 1 Introduction

Craniospinal irradiation is delivered in two or three partial treatment plans with different craniocaudal isocentre locations because of the long treatment target. Different methods for optimising the fields at the junction region exist [1], [2], [3], [4]. In conventional treatments utilising narrow field junctions, the craniocaudal field localisation is often based on the skull position. The isocentre distance should not be altered even if the spinal length or position during treatment delivery was different from the treatment plan [5]. Thus, if the patient is taller than in the treatment planning computed tomography (CT) scan, the most caudal part of the target is underdosed, and if the patient is shorter than in the planning CT image, the healthy tissue caudal to target is overdosed. In techniques utilising wide field junctions and low dose gradients, the craniocaudal isocentre distance may be slightly adjusted to the patient position [5], [6]. However, systematic craniocaudal isocentre distance deviations >3 mm have been reported to cause significant dosimetric deviations even with low dose gradient techniques [4], [7], [8], [9].

Positioning a patient for craniospinal irradiation is complicated due to the long treatment volume with non-rigid anatomy. Diurnal variation in spinal length might affect the setup accuracy. Several groups have reported that the human height decreases by 5–7 mm between morning and afternoon in average [10], [11], [12]. Two studies reported an average height gain of 19–20 mm during overnight sleep [13], [14]. However, 54% of the height gain was lost during the first hour after rising up [13]. The diurnal variation is mostly explained by compression of the intervertebral discs in upright position [15], [16], [17], [18]. The spinal length variation might lead to decreased setup accuracy in craniospinal irradiation if the treatment is delivered at a different time of the day than the planning CT image. Recently, a median decrease of −1.0 mm in distance between the eleventh thoracic vertebra and fourth lumbar vertebra over the day was observed in children receiving radiotherapy for multiple sites [19].

This study aimed to quantify the correlation between the treatment fraction timing and the distance between the base of skull and the third lumbar vertebra (L3) in craniospinal irradiation. These locations were selected because they were the most cranial and caudal landmarks visible in the setup images of all patients. To our knowledge, this is the first quantification of diurnal variation in spinal length comprising almost the whole spine and including both adult and paediatric radiotherapy patients.

## 2 Materials and methods

Planning and setup images of 13 craniospinal irradiation patients were retrospectively collected. The study was approved by Wellbeing Services County of Pirkanmaa (research permit number R21663). The patients were treated in 10–22 fractions, with a total of 224 fractions. One fraction was excluded because of missing images. The age range of the patients was 3–54 years and the median age was 17 years.

The patients were treated in supine position. They were fixated using a thermoplastic mask and a vacuum bag (n=8), knee rest (n=4) or arm support straps (n=1). Four paediatric patients who were treated under anaesthesia were fixated similarly except for an opening in their thermoplastic masks to accommodate the anaesthesia masks. Planning CT images were acquired with 3 mm slice thickness, except for one patient scanned with 1 mm slice thickness. The scan range was from the top of the head to the hips.

The initial setup was based on the treatment room lasers. Next, two-dimensional kilovoltage setup images were acquired at the skull, upper spine and lower spine. The field-of-view was 26.5 × 20.0 cm. The skull image included the skull, and, in some patients, the cervical spine. The cranial edge of the lower spine image was located between the eighth thoracic and second lumbar vertebra and the caudal edge between the third and fifth lumbar vertebra. For seven patients treated using volumetric modulated arc therapy, orthogonal images were acquired. For these patients, the craniocaudal offsets were determined as the average of the deviation in lateral and anterior-posterior images. For six patients treated using three-dimensional conformal radiotherapy, only lateral skull images and anterior-posterior spine images were acquired. Translational setup errors were corrected by shifting the treatment couch. Repeated setup images were acquired only if manual repositioning was required due to deformational or rotational errors. For the patients treated using three-dimensional conformal radiotherapy, the anterior-posterior offset of the skull and the lateral offset of the spine were applied for the full target length.

**Fig. 1:**
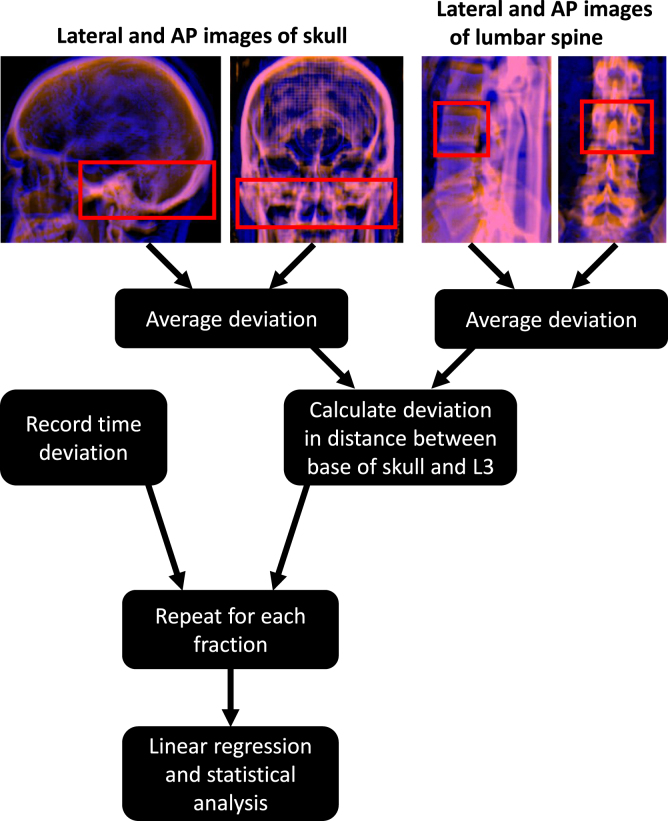
The data analysis workflow. In the top images, the lateral and anterior-posterior (AP) digitally reconstructed radiographs (red) and setup images (blue) are overlaid. The red boxes indicate the match volumes, which were placed at the base of the skull and the third lumbar vertebra (L3), and used for determining the setup deviation in craniocaudal direction. (For interpretation of the references to colour in this figure legend, the reader is referred to the web version of this article.)

The last images acquired before treatment delivery were used for determining the skull-to-L3 distance deviations. The initial couch positions of the skull and lower spine setup images in craniocaudal direction were recorded. The setup images were matched to the digitally reconstructed radiographs using ARIA Offline Review v. 16.1 (Varian Medical Systems, Palo Alto, California, USA). Automatic 2D/2D image match was used initially, and necessary corrections were made manually. The regions of interest were the base of the skull and L3 (Fig. 1). The registration was based on bony structures. The image match results were added to the initial couch positions, resulting in a setup-corrected couch position. Next, the setup-corrected couch position of the skull image was subtracted from that of the lower spine. The resulting value was compared to the initial couch position difference in the treatment plan. The times of planning CT image acquisition and beginning of each fraction were collected. The time deviation was defined as the difference between the times of the day when the treatment fraction started and when the planning CT image was acquired.

Statistical analysis was conducted using Matlab v. R2018b (The MathWorks, Inc., Natick, Massachusetts, United States). The setup error versus time deviation data was fitted using linear regression. Pairwise two-tailed t-test was used to test if there was a statistically significant difference between the setup errors of fractions delivered at different time intervals compared to the imaging session.

## 3 Results

**Fig. 2:**
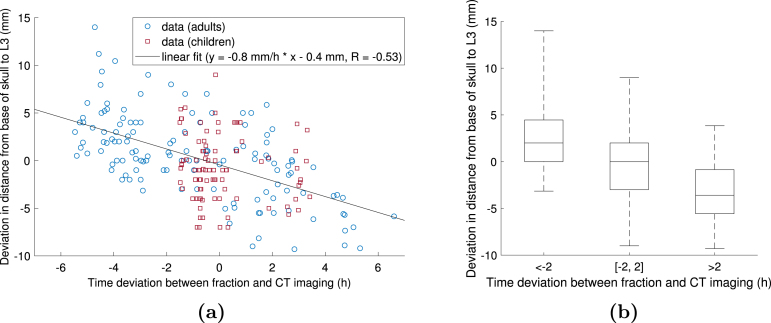
Figure (a) demonstrates the effect of time deviation between the treatment fraction and the computed tomography (CT) imaging session on the deviation in distance between the base of the skull and the third lumbar vertebra (L3) in 223 craniospinal irradiation treatment fractions. The data points of adult (age ≥ 15 years) and paediatric patients (age < 15 years) are shown in blue circles and red squares, respectively. The solid line represents the linear fit to all data points. Figure (b) demonstrates the distribution of the distance deviations in fractions delivered more than two hours before the imaging session, less than two hours apart from the imaging session and more than two hours later than the imaging session. The top and bottom of the box denote the 75th and 25th percentile, respectively, the line inside the box denotes the median value, and the whiskers denote the minimum and maximum value. There was a statistically significant pairwise difference (p<0.05) between each of the groups.

A moderate correlation between the deviation in skull-to-L3 distance and the time deviation was observed (p<0.001, R=−0.53), as shown in Fig. 2(a). The slope of the regression line was −0.8 mm/h (95% confidence interval (CI) [−1.0, −0.7] mm/h). The intercept was −0.4 mm (95% CI [−0.9, 0.0] mm).

In adult patients (age≥15 years, n=8), who were not treated under anaesthesia, the time deviations were evenly distributed between −5.5 and ＋6.6 h. Paediatric patients (age<15 years, n=5) were treated with <2 h deviation between the times of the day of the daily fraction and the planning CT image in 82% of fractions due to anaesthesia requirements for four of them. In adults, the correlation was stronger than in the total patient population (slope = −0.9 mm/h, R=−0.62). In children, the correlation was negligible (slope = −0.3 mm/h, R=−0.12).

The regression slopes and correlation coefficients of data from individual patients had lower average magnitudes than those calculated for all patients. In adults, the median (range) slopes and correlation coefficients were −0.3(−1.3, −0.1) mm/h and −0.27(−0.62, −0.06), respectively. In children, the median (range) slopes and correlation coefficients were −0.2(−1.6, 2.6) mm/h and −0.10(−0.36, 0.28).

The distance deviation in fractions delivered >2 h earlier than the time of the day of the planning CT image was 2.8±3.6mm (mean±standard deviation, n=57). The distance deviation in fractions delivered within 2 h of the time of the day of the planning CT image was −0.2±3.7mm (n=127). The distance deviation in fractions delivered >2 hours later than the time of the day of the planning CT image was −3.3±3.1mm (n=39). There was a statistically significant (p<0.001) pairwise difference between each group, as demonstrated in Fig. 2(b). A similar plot with 1 h steps is shown in Supplementary Figure S1.

## 4 Discussion

In this study, we observed a moderate correlation between the skull-to-L3 distance and the time deviation between the treatment fraction and planning CT image in craniospinal irradiation. The distance decreased by 0.8 mm/hour in average, which is similar to earlier studies reporting the diurnal height variation [10], [11], [12].

We measured the distance between the base of skull and L3 because those are typical isocentre locations in craniospinal irradiation. Furthermore, L3 was the lowest vertebra visible in setup images of all patients. In three-isocentre treatment plans, the distance deviation would be divided between two junction regions. The setup errors may not be evenly distributed between the junction regions [6]. In our experience, small deviations in the cervical spine position may cause setup errors not related to the diurnal height variation at the lumbar spine.

We have previously simulated the dosimetric effect of craniocaudal isocentre distance deviations using the same patient data as in this study with a wide-junction partial-arc volumetric modulated arc therapy technique [6]. We reported that the fraction-specific near-maximum and near-minimum doses of the junction region changed by <5 percentage points when the isocentre distance deviation was ≤3 mm except for one fraction. Our results were similar to earlier studies simulating the effect of isocentre distance deviations [4], [7], [8], [9]. However, the dosimetric consequences of spinal length deviations depend on the planning, treatment and positioning techniques. In this study, the average distance deviations between the base of skull and L3 in fractions delivered more than 2 h earlier and later than the imaging session were approximately ＋3 mm and −3 mm, respectively. Therefore, we advise scheduling craniospinal irradiation with <2 h deviation between the times of the day of the daily fraction and planning CT image. This advice is based on dosimetric results of earlier studies on wide-junction volumetric modulated arc therapy treatments. In narrow-junction treatment techniques, similar if not smaller time deviation is expected to be required because even the smallest isocentre distance deviations could cause considerable overdosing or underdosing of the junction region.

In our study, children were in most cases treated with <2 h time deviation compared to the planning CT image due to the schedules of the anaesthesia team. The negligible correlation between the paediatric patients’ skull-to-L3 distance deviation and time deviation is probably caused by the small time deviations and not by inherent differences between children and adults. According to Meijer et al. [19], there was no significant correlation between height or weight and the diurnal variation of spinal length in paediatric patients, which might indicate that the patients’ size or age does not affect the spinal length deviations in radiotherapy.

In addition to the diurnal height variation, there are several other factors causing setup errors in craniospinal irradiation. This, and the low number of data points per patient may explain the low correlation coefficients and slopes of the regression line in individual patients. Discomfort, poor cooperation, incorrect neck angle and suboptimal fixation method might also cause setup errors. In our study, some patients were fixated using full-body vacuum bags and some without them. We were not able to compare these patient groups because of the small number of patients in the no-vacuum-bag group and different distributions of time deviations. The effect of fixation on the setup accuracy would require further research with a larger patient population. Timing the treatment fractions accurately to correspond to the time of the day when the planning CT image was acquired does not completely eliminate the isocentre distance deviations. However, according to our results, it does reduce the average deviations.

The body-mass-index, sleep duration and physical activity have been reported to impact the diurnal height variations [12], [17], [18]. Furthermore, the height loss is not linear but most of it happens in the morning [10], [13]. Due to the retrospective nature of this study, we were not able to control these factors. Another limitation was the relatively low number of patients.

A strength of our study was that we had access to image data of almost the whole spine. While Meijer et al. [19] focused on paediatric patients, our data included both adults and children. The conclusions of our study might also be valuable for other radiotherapy targets including the whole spine, such as total marrow irradiation [20].

In conclusion, we report an average decrease of 0.8 mm/hour in the skull-to-L3 distance measured from the setup images of craniospinal irradiation patients. Diurnal changes might have a significant impact on dosimetry; therefore, timing the treatment fractions within two hours of the planning imaging session is advisable. Dosimetric analyses are still warranted for a final recommendation.

## CRediT authorship contribution statement

**Annele Heikkilä:** Conceptualization, Methodology, Formal analysis, Writing – original draft, Visualization, Funding acquisition. **Maija Rossi:** Conceptualization, Writing – review & editing, Project administration. **Antti Vanhanen:** Conceptualization, Writing – review & editing , Project administration. **Tuomas Koivumäki:** Conceptualization, Writing – review & editing, Supervision. **Michiel Postema:** Writing – review & editing, Supervision, Funding acquisition. **Eeva Boman:** Conceptualization, Writing – review & editing, Supervision.

## Funding

This study was financially supported by the Support Foundation of Tampere University Hospital, Project No. MK351, the Vilho, Yrjö and Kalle Väisälä Foundation, and the Academy of Finland , Grant No. 340026.

## Declaration of competing interest

The authors declare that they have no known competing financial interests or personal relationships that could have appeared to influence the work reported in this paper.

## Appendix A Supplementary data

MMC S1 Boxplot illustrating skull-to-L3 distance deviations at different time intervals compared to planning image acquisition.
