# Supplementary material for: Diurnal variation of the distance between cranium and the third lumbar vertebra and its implications for craniospinal irradiation
Source: Phys Imaging Radiat Oncol. 2025 Apr 8;34:100760. doi: 10.1016/j.phro.2025.100760 (PMC12036028; doi:10.1016/j.phro.2025.100760)
Supplement: MMC S1 — Boxplot illustrating skull-to-L3 distance deviations at different time intervals compared to planning image acquisition. [file mmc1.pdf]

Supplementary material

to "Diurnal variation of the distance between cranium and the third lumbar vertebra and its implications for craniospinal irradiation" by Annele Heikkilä, Maija Rossi, Antti Vanhanen, Tuomas Koivumäki, Michiel Postema, Eeva Boman

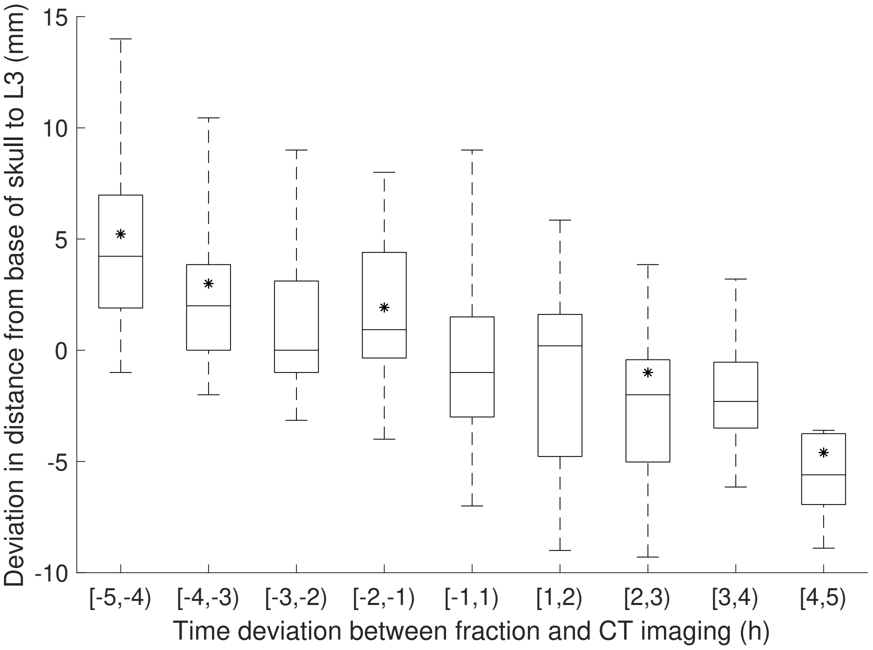

Figure S1: Distribution of the distance deviations between base of skull and third lumbar vertebra (L3) in fractions delivered at different time intervals compared to the computed tomography (CT) imaging session. The top and bottom of the box denote the 75th and 25th percentile, respectively, the line inside the box denotes the median value, and the whiskers denote the minimum and maximum value. Asterisk inside the box indicates that the deviation in the group was significantly different ($p < 0.05$) from the group with time deviation between -1 and 1 hours. Fractions with time deviation higher than ±5 hours were excluded from the figure due to a low number of data points.
